# Identification of Novel miRNAs and miRNA Dependent Developmental Shifts of Gene Expression in *Arabidopsis thaliana*

**DOI:** 10.1371/journal.pone.0010157

**Published:** 2010-04-13

**Authors:** Shuhua Zhan, Lewis Lukens

**Affiliations:** Department of Plant Agriculture, University of Guelph, Guelph, Ontario, Canada; King Abdullah University of Science and Technology, Saudi Arabia

## Abstract

microRNAs (miRNAs) are small, endogenous RNAs of 20∼25 nucleotides, processed from stem-loop regions of longer RNA precursors. Plant miRNAs act as negative regulators of target mRNAs predominately by slicing target transcripts, and a number of miRNAs play important roles in development. We analyzed a number of published datasets from *Arabidopsis thaliana* to characterize novel miRNAs, novel miRNA targets, and miRNA-regulated developmental changes in gene expression. These data include microarray profiling data and small RNA (sRNA) deep sequencing data derived from miRNA biogenesis/transport mutants, microarray profiling data of mRNAs in a developmental series, and computational predictions of conserved genomic stem-loop structures. Our conservative analyses identified five novel mature miRNAs and seven miRNA targets, including one novel target gene. Two complementary miRNAs that target distinct mRNAs were encoded by one gene. We found that genes targeted by known miRNAs, and genes up-regulated or down-regulated in miRNA mutant inflorescences, are highly expressed in the wild type inflorescence. In addition, transcripts upregulated within the mutant inflorescences were abundant in wild type leaves and shoot meristems and low in pollen and seed. Downregulated transcripts were abundant in wild type pollen and seed and low in shoot meristems, roots and leaves. Thus, disrupting miRNA function causes the inflorescence transcriptome to resemble the leaf and meristem and to differ from pollen and seed. Applications of our computational approach to other species and the use of more liberal criteria than reported here will further expand the number of identified miRNAs and miRNA targets. Our findings suggest that miRNAs have a global role in promoting vegetative to reproductive transitions in *A. thaliana*.

## Introduction

Plant microRNAs (miRNAs) are involved in multiple developmental and physiological processes and negatively regulate gene transcript abundance through post-transcriptional repression of mRNAs, primarily by target cleavage [1]. MiRNAs are generated from endogenous loci that produce transcripts with internal stem loop structures that are processed to 20∼25nt small double stranded RNAs. Many proteins of the miRNA biogenesis pathway are known. RNA polymerase II generates pri-miRNAs which are stabilized by DAWDLE (DDL) [2]. Pri-miRNAs are converted to stem-loop pre-miRNAs by DICER-LIKE1 (DCL1) [3] which interacts with the double stranded RNA-binding protein HYPONASTIC LEAVES1 (HYL1) [4] and SERRATE (SE) [5]. The pre-miRNAs are processed either to 21 nt mature miRNA/miRNA* duplexes by DCL1 [4] or to 24 nt mature miRNA duplexes by DCL3 [6]. The mature miRNA duplexes are methylated by the S-adenosyl methionine-dependent methyltransferase HUA ENHANCER1 (HEN1) [7]. HASTY (HST) is the plant homolog of mammalian EXPORTIN 5 which is known to export pre-miRNA [8]. One strand of the methylated miRNA/miRNA* duplex is preferentially assembled with the ARGONAUTE1, AGO1 protein, which slices the mRNA targets at the miRNA target site [9]. Despite the existence of this core pathway, its mutants have different effects on miRNA abundance and miRNA target abundance. Hypomorphic *dcl1* mutants have undetectable or very low levels of mature miRNAs [10], and miRNA accumulation is similarly undetectable or very weak in null *hen1* and *hyl1* mutants [11], [12]. Although HST is frequently depicted as an exportin [1], its function has not been validated, and *hst* mutants fail to accumulate miRNAs in the nucleus [13]. In *hst* mutants, some miRNAs accumulate to wild-type levels, others have reduced abundance, and others are not detectable [13].

Over 184 *Arabidopsis* miRNAs have been identified (miRBASE release 10.0) [14]. miRNAs have been predicted to regulate the expression of more than 600 genes [15], and 225 genes are known targets [16]. miRNAs and their targets have been discovered using approaches including cloning and sequencing sRNAs [10], [17], bioinformatic prediction from genomic sequences [18], and sequencing cleaved mRNAs by parallel analysis of RNA ends (PARE) [19]. Utilizing miRNA biogenesis/transport mutants for miRNA and miRNA target identification has not been widely employed. First, many miRNA target transcripts do not accumulate when their cognate miRNAs decrease. In one study of *hen1* and *dcl1* mutants, miRNA target abundances were greater than wild type in eight of eight known targets examined [12]; while in another study, five of ten transcripts were significantly up-regulated [20]. Two of three miRNA targets accumulate within the *hst-1* mutant [13], and five of eight target transcripts had higher abundance in the *hyl1* mutant compared to wild-type [12]. Second, a large number of genes are misexpressed in individual mutants causing an abundance of upregulated genes that are not miRNA targets [21], [22].

The core function of plant miRNAs is in the regulation of development [23]. Hypomorphic *dcl1* mutants and null *hyl1*, *hst*, and *hen1* mutants reveal both shared and distinct developmental defects that may represent misexecution of developmental transitions [24]. All canonical 21nt miRNA mutants (*dcl1*, *hen1*, *hst*, and *hyl1*) have a shorter stature, delayed vegetative to reproductive transitions, and reduced female fertility compared to wild type [5], [12], [25], [26]. *Hen1* mutants have defects in floral meristem identity, a trait also shared by *dcl1* partial loss-of-function mutants [11], [25], and the juvenile development phase of *hst15* is shortened [26]. The effects of many individual misexpressed miRNA targets also highlight the role of miRNAs in plant development. Misexpression of miRNA target genes leads to plant developmental defects in meristems (HD-ZIPIII genes) [27], leaves (TCP) [28], flowers (AP2 genes; CORNGRASS1) [29], [30], and roots (NAC1) [31]. Misexpression of targets also affects plant vegetative phase changes [5], [32]. Despite these phenotypic observations, to our knowledge it is unknown if disrupting plant miRNAs can cause one tissue's transcriptome to resemble that of a different tissue. For example, in animals, delivering miRNAs into cells can cause their transcriptomes to resemble the transcriptome of the tissue where the miRNA is preferentially expressed [33].

This study performs novel analyses on a number of published data sets to identify miRNAs and their targets and to identify miRNA dependent tissue biases within the transcriptome. We integrate data sets including microarray profiling and sRNA deep sequencing data from miRNA biogenesis/transport mutants, microarray profiling data of mRNAs in a developmental series, and computational predictions of conserved, genomic stem-loop structures. Our results show that known miRNA targets fail to explain transcriptome patterns amongst miRNA mutants. Interestingly, transcript changes in *hyl1* and *hst* mutants greatly overlapped, suggesting a novel functional connection between HST and HYL. Transcript changes in *dcl1* and *hyl1* were surprisingly distinct, and *dcl3* had little effect on the transcriptome. We identified novel miRNA targets and five mature miRNAs, four of which had high similarity to previously identified mature miRNAs. One miRNA gene encodes two complementary miRNAs that target distinct transcripts. These miRNAs target transcription factors, and a gene with 4-α-glucanotransferase activity, thereby expanding miRNA functionality in metabolism. Finally, we found that genes regulated by miRNAs show strong tissue-specific patterns of expression. miRNA regulated genes tend to be highly expressed in the inflorescence, and miRNAs cause the inflorescence transcriptome to both diverge from a meristem and leaf-like state and to acquire a pollen and seed-like state.

## Results

### Known miRNA targets do not explain miRNA-defective mutant transcriptome patterns

**Figure 1:**
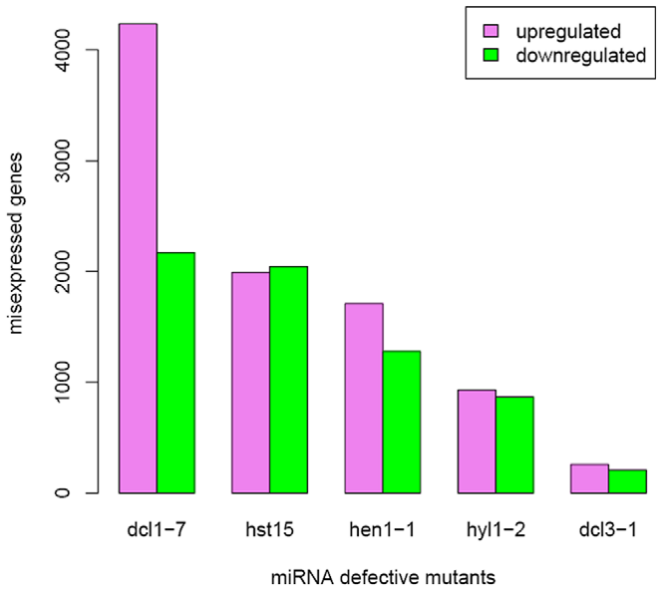
Numbers of transcripts that were up-regulated or down-regulated in mutant lines compared to wild-type lines. Genes were classified as up-regulated or down-regulated if their transcript abundance increased or decreased, respectively, and the changes were statistically significant (FDR = 0.05).

**Figure 2:**
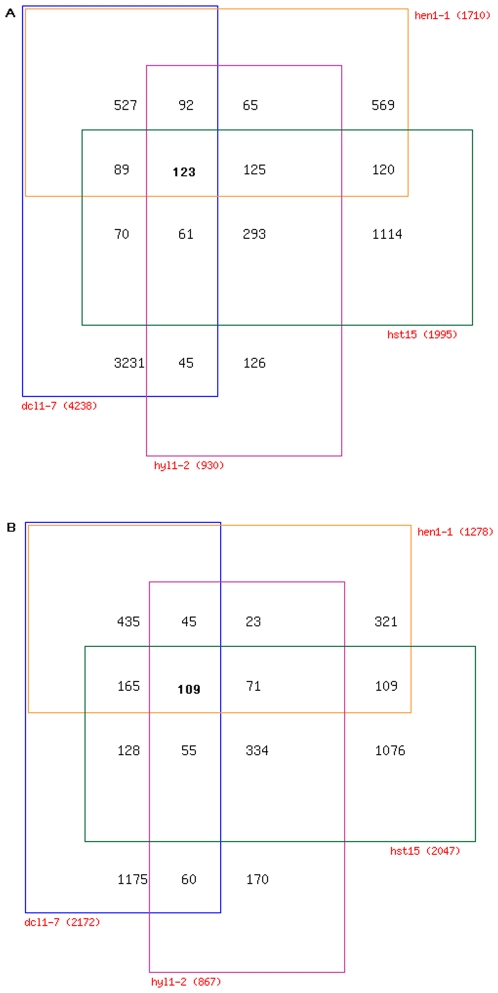
Venn diagrams showing up- and down-regulated genes across the mutants *dcl1-7*, *hen1-1*, *hyl1-2* and *hst15*. (A) Among up-regulated genes, 123 genes were shared among the miRNA mutants. 117 of the 123 genes have no cross-hybridization and were chosen for further study. (B) Among down-regulated genes, 109 genes are shared among the miRNA mutants. 105 of the 109 genes have no cross-hybridization and were chosen for further study.

We first analyzed ATH1 gene expression data from the inflorescences of the miRNA-defective mutants, *dcl1-7*, *dcl3-1*, *hen1-1*, *hyl1-2* and *hst15* [21], [34] (Figure 1). The numbers of known, up-regulated miRNA targets and the levels to which known miRNA targets were up-regulated were both poorly correlated with the number of misexpressed transcripts across the canonical miRNA mutants. We found that probe sets homologous to 131 of 225 known miRNA targets are present on the ATH1 microarray, excluding cross hybridizing probe sets. The number of known, upregulated miRNA targets in *hst15* (29) was less than the number in other canonical mutants (44 in *dcl1*; 37 in *hyl1*; and 38 in *hen1*; Figure S1), although 1,995 genes are upregulated in the *hst15* mutant (Figure 1). The log-fold change distributions of known miRNA targets were also very similar between mutants with large transcriptome changes and mutants with small transcriptome changes (Figure S2). Differences and similarities of transcriptome changes between mutants were also not explained by known miRNA targets, as the number of known miRNA targets shared between two mutants was not correlated with the number of upregulated genes shared between two mutants. Of the 930 up-regulated genes in *hyl1-2*, 65% of transcripts increased in *hst15*, 35% in *dcl1-7*, and 44% in *hen1-1* (Figure 2A). Of the 1,995 up-regulated genes in *hst15*, 30% transcripts increased in *hyl1-2*, 17% in *dcl1-7*, and 12% in *hen1-1* (Figure 2A). *hst* and *hyl1* shared 18 known miRNA targets, the same number as *hst15* and *dcl1-7* and less than between *hen1* and *hyl1* (24) (Figure S3). Likewise, *dcl1* and *hen1* have similar transcriptomes- of the 1,710 up-regulated genes in *hen1-1*, 49% of the transcripts increased in *dcl1-7* (Figure 2A), and of the 4,238 up-regulated genes in *dcl1-7*, 20% of the transcripts increased in *hen1-1* (Figure 2A). However, the known targets' overlap between *dcl1-7* and *hen1-1* (22) was about as great as those between *dcl1-7* and *hyl1-2* (24), and only slightly more than the overlap between *dcl1-7* and *hst15* (18) (Figure S3).

These analyses also revealed two unexpected results. First, the similarity of transcript abundance changes in *hst15* and *hyl1-2*, and the differences between *dcl1* and *hyl1* were unexpected given the predicted miRNA biogenesis/transport pathway [1]. The high level of *hst15* and *hyl1-2* intersection was described above. In contrast, 35% of the transcripts that increased in *hyl1* increased in *dcl1-7*, and 8% of the transcripts that increased in *dcl1* also increased in *hyl1* (Figure 2A). The down-regulated genes showed similar intersections (Figure 2B). Second, disrupting 21nt miRNA biogenesis had a severe effect on the plant transcriptome; while disrupting long miRNA biogenesis and siRNA biogenesis had a small effect. The number of genes with transcript levels that increased relative to wild type controls ranged from 930 to 4,238 among the canonical 21nt miRNA biogenesis mutants *dcl1-7*, *hen1-1*, *hst15*, and *hyl1-2* (Figure 1). The number of transcripts that decreased ranged from 867 to 2,172. In contrast, 258 genes increased in abundance and 210 genes decreased in abundance in the *dcl3-1* mutant (Figure 1).

### Computational prediction of novel miRNAs and targets

Our results suggested that known miRNA targets fail to explain transcription patterns amongst miRNA mutants, and we devised a method to identify both novel miRNAs and miRNA targets. The ATH1 array contains approximately 22,750 probe sets representing 23,750 genes. We reasoned that the transcripts that changed within the mutants included mRNAs to which miRNAs bind and cleave and downstream transcripts influenced by these direct targets. To identify novel miRNAs and miRNA targets, we defined a core set of genes up-regulated across the *dcl1-7*, *hen1-1*, *hyl1-2* and *hst* mutants. This set contains 117 genes (123 probe sets changed significantly across all mutants (Figure 2A), but six probe sets cross-hybridized with multiple genes and were removed).

Transcription factors are over-represented among known miRNA targets [35]. To infer if our core set likely contained target genes, we evaluated the frequency of GO SLIM gene ontology terms within the set. Transcription factors occurred twice as frequently as expected (12% vs. 6%), (Figure S4; Table 1; Fisher's Exact Test P<0.001), and the biological process “transcription” was over-represented within the up-regulated genes (6% vs. 4%, Table S1). Interestingly, genes classified in the “response to abiotic or biotic stimulus” biological process were also more frequent within the up-regulated genes as compared to the non-upregulated genes (6% vs. 3%, Table S1), and genes with unknown molecular functions occurred at less than half the expected frequency among up-regulated genes (11% vs. 24%, Table 1). Genes without GO SLIM terms were not inherently less likely to change in abundance than genes with GO SLIM terms, because the number of genes with unknown molecular functions among down-regulated genes is similar to their genome proportion (Table 1). Among the 105 genes significantly down-regulated across the mutants, there was no evidence that molecular function classes or biological processes were over or under represented (Table 1; Table S1; Fisher's Exact Test P>0.01).

**Table 1:** Goslim categories in molecular function (MF) of differentially expressed genes and non-differentially expressed genes in *dcl1-7*, *hen1-1*, *hyl1-2 and hst15*.

	Non-up-regulated genes		Up-regulated genes		Down-regulated genes		Non-down-regulated genes	
Goslim categories	Genes	Proportion (%)	Genes	Proportion (%)*	Genes	Proportion (%)†	Genes	Proportion (%)
DNA or RNA binding	1479	6.36	4	3.03	7	5.43	1476	6.34
hydrolase activity	2107	9.06	17	12.88	18	13.95	2106	9.05
kinase activity	1174	5.05	7	5.30	2	1.55	1179	5.07
nucleic acid binding	431	1.85	3	2.27	1	0.78	433	1.86
nucleotide binding	1000	4.30	2	1.52	2	1.55	1000	4.30
other binding	2127	9.14	19	14.39	17	13.18	2129	9.15
other enzyme activity	2225	9.56	16	12.12	13	10.08	2228	9.57
other molecular functions	699	3.00	2	1.52	4	3.10	697	3.00
protein binding	1775	7.63	5	3.79	12	9.30	1768	7.60
receptor binding or activity	181	0.78	1	0.76	1	0.78	181	0.78
structural molecule activity	385	1.65	1	0.76	1	0.78	385	1.65
transcription factor activity	1358	5.84	16	12.12	10	7.75	1364	5.86
transferase activity	1664	7.15	15	11.36	10	7.75	1669	7.17
transporter activity	1028	4.42	9	6.82	1	0.78	1036	4.45
unknown molecular functions	5635	24.22	15	11.36	30	23.26	5620	24.15

*Proportions in the 15 Goslim MF categories differed significantly from those for non-up-regulated genes (Fisher's exact test, P = 2.4×10^−4^).

† Proportions in the 15 Goslim MF categories did not differ significantly from those for non-down-regulated genes (Fisher's exact test, P = 0.1619).

The core set of 117 genes up-regulated across all four canonical miRNA biogenesis mutants represented a conservative starting point to identify novel miRNAs and targets. We searched for high complementarity between a set of 1,984 sRNA sequences generated by deep sequencing of sRNAs from the *A. thaliana rdr2-1* mutant [10] and 158 predicted mRNAs derived from the 117 up-regulated genes, allowing for three or fewer mismatches. We identified 113 sRNAs complementary to 76 putative mRNAs that were derived from 56 of the 117 genes. We ran a BLAST analysis of the 113 sRNAs against 2,585 conserved *Arabidopsis* 20mers that have homology to conserved genomic loci that could generate a hairpin structure (*At*Set3) [18]. Forty of the 113 sRNAs were perfectly matched by 20mers in *At*Set3. Twenty-nine were previously characterized miRNAs. From the remaining eleven sRNAs, we identified five novel, mature miRNA sequences. Three of the five mature miRNAs (miRNAs 3, 4, and 5) are encoded by two MIR169 genes on chromosomes 3 and 5 (Table 2). MiRNA 3 is novel. The other mature miRNAs differ from the mature miRNAs in miRBase entries MIR166a, MIR169a, MIR169b, MIR169f and MIR169g by between one or two nucleotides. All candidate miRNAs were expressed in the *rdr2* mutant and were not present in sequenced sRNAs from the *dcl1* mutant (Table 3). Only miRNA 2 was found in wild type. These miRNAs have low abundance (Table 3), but the abundance is not unusual relative to other known miRNAs. Lu et al. found that 65% (30 out of 46) of known miRNAs have a count equal or less than six out of 4,573 sRNAs sequenced in a 454 analysis of the *rdr2* mutant inflorescence [10].

**Table 2:** Newly identified mature miRNAs in *Arabidopsis*.

miRNA identifier	miRNA gene	Chr.	Coordinates	Arm	Mature miRNA sequence
*1*	*MIR169f*	3: [−]	4805723–4805825	5′	GAGCCAAGGAUGACUUGCCGG
	*MIR169g*	4: [−]	11483045–11483125	5′	GAGCCAAGGAUGACUUGCCGG
*2*	*MIR166a*	2: [+]	19183197–19183330	3′	UUCGGACCAGGCUUCAUUCCC
*3*	*MIR169a-3p.1*	3: [−]	4359017–4359211	3′	GCAAGUUGUCCUUGGCUACA
*4*	*MIR169a-5p.1*	3: [−]	4359017–4359211	5′	UGCAGCCAAGGAUGACUUGCC
	*MIR169b.1*	5: [+]	8527512–8527616	5′	UGCAGCCAAGGAUGACUUGCC
*5*	*MIR169a-5p.2*	3: [−]	4359018–4359210	5′	GCAGCCAAGGAUGACUUGCCG
	*MIR169b.2*	5: [+]	8527513–8527615	5′	GCAGCCAAGGAUGACUUGCCG

Newly identified miRNAs are listed. The predicted stem-loop of miRNA is transcribed from the forward strand (+) or reverse strand (−) of the annotated chromosome (Chr.) from start to end coordinates (Coordinates). The mature miRNA is generated from the arm of the predicted stem-loop (Arm).

**Table 3:** Novel miRNA expression (454 raw values) in sRNA mutants and wild-type inflorescences*.

miRNA identifier	rdr2-1	rdr6-15	Col-0	dcl1-7	dcl234
1	1	0	0	0	0
2	6	1	4	0	14
3	2	0	0	0	0
4	1	0	0	0	0
5	1	0	0	0	0
Total sRNAs†	4573	6441	7488	8663	6214

*Counts of putative miRNAs were made from 454 sRNA sequence data as described in GEO dataset GSE5343 (Lu et al. 2006).

† This row shows the total number of genome-matching small RNAs sequenced in each 454 library.

**Figure 3:**
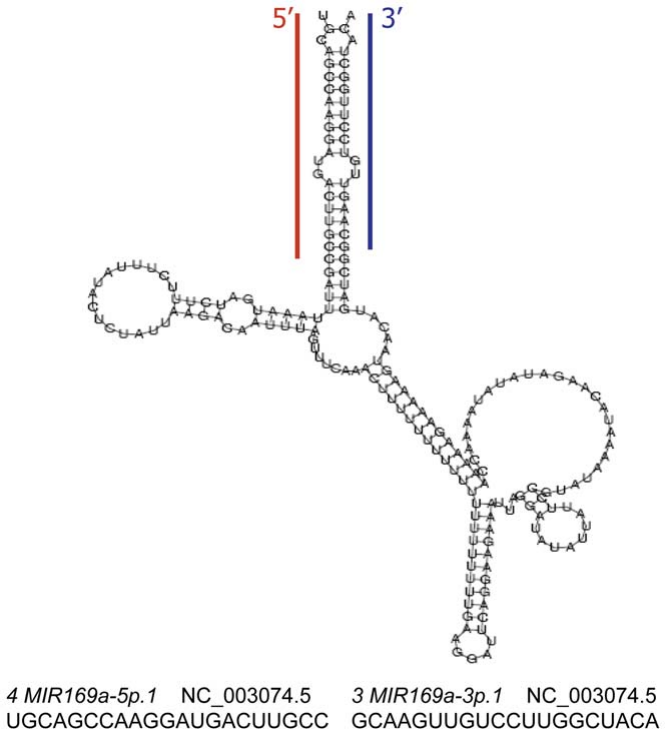
Predicted hairpin structure of miRNAs 3 and 4. miRNA 4 (red bar) and miRNA 3 (blue bar) target different genes and are encoded by complementary sequences within the stem-loop precursor.

Pre-miRNA secondary structures were predicted by RNAfold [36] and evaluated by MIRcheck [18]. The predicted secondary structures are shown in Figure S5. Interestingly, miRNAs 3 and 4 have homology to complementary sequences in the same stem loop of MIR169a (Figure 3). One miRNA has high complementarity with the disproportionating enzyme (DPE2; *At2g40840*), which has 4-α-glucanotransferase activity and is an essential component of the pathway from starch to sucrose in leaf cells [37]. *At2g40840* is relatively highly abundant in leaves, meristems and developing siliques and less abundant in pollen, seeds, and siliques in late developmental stages. The other miRNA has high complementarity with two CCAAT-binding transcription factors (*At1g17590*, *At1g54160*). The mature miRNAs 1, 2, 4, and 5 have high complementarity to transcription factor encoding mRNAs targeted by MIR166 and MIR169 miRNAs [18], [21] (Table 4); however mature miRNAs 2, 4 and 5 have predicted novel target cleavage sites within these transcripts. We also observed that the CCAAT binding transcription factor *At1g54160* is targeted by different mature miRNAs (1, 4, and 5). These findings revealed that one miRNA can regulate multiple target genes, and one gene can be targeted by multiple miRNAs.

**Table 4:** Predicted miRNA targets.

miRNA identifier	miRNA family	Target protein class	Target genes
1	MIR169	CCAAT-binding transcription factor	*At1g54160,At3g05690,At5g06510*
2	MIR166	HD-Zip transcription factors	*At1g30490,At5g60690*
3	MIR169	4-alpha-glucanotransferase activity	*At2g40840*
4	MIR169	CCAAT-binding transcription factor	*At1g17590,At1g54160*
5	MIR169	CCAAT-binding transcription factor	*At1g17590,At1g54160*

### Tissue specific transcriptional patterns of miRNA regulated genes

**Figure 4:**
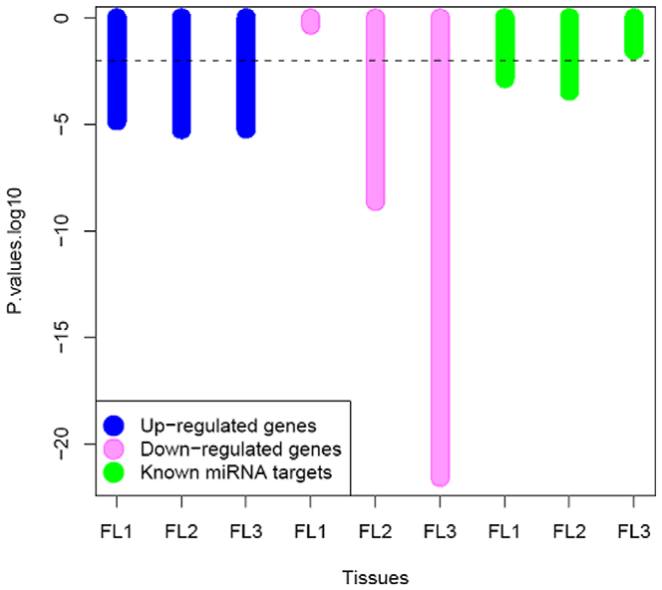
Known miRNA targeted genes, and genes up or down-regulated across mutants were highly abundant within the inflorescence. The inflorescence expression ranks of genes within these groups were significantly higher than ranks of genes outside these groups. Log (base 10) P values are plotted for three inflorescence tissues (FL1, FL2, and FL3). The dashed line shows an α critical value of 0.01.

We tested if genes mis-expressed in miRNA mutants have tissue-specific patterns of expression. To determine this, we computed the ranks of the genes up-regulated and down-regulated in the canonical miRNA mutant inflorescences across 23 tissues. Tissues included roots, meristems, leaves, flowers, siliques, seeds, and mature pollen [38]. If genes up- or down-regulated across mutants had high or low transcript abundances within a tissue, these genes had high or low ranks within that tissue, respectively. We also ranked the transcripts that did not change in abundance amongst the mutants in each of the 23 tissues to obtain the expected distribution of gene expression ranks within each tissue. Significant differences in rank distributions were tested using the Komolgorov-Smirnov (KS) test (see materials and methods). We found that up-regulated core set genes were significantly abundant in the inflorescence relative to other tissues (Figure 4). This finding suggested that known miRNA targets would also be highly abundant within the inflorescence. Indeed, known targets were highly abundant within the inflorescence relative to other tissues (Figure 4). Interestingly, the genes down-regulated in the mutants were also highly expressed in the inflorescence relative to other tissues (Figure 4).

**Figure 5:**
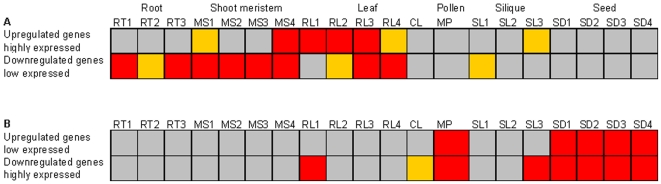
Tissue specificity of genes up- and down-regulated in miRNA biogenesis mutant inflorescences. Grey, yellow and red indicate no significance, significance at P<0.05, and significance at P<0.01 respectively. (A) Tissues in which genes up-regulated in the mutants had high abundance, and tissues in which genes down-regulated in the mutants had low abundance. Colors in the top row indicate if genes up-regulated in the mutants are expressed at a significantly high level in the wild type tissue. Colors in the bottom row indicate if genes down-regulated in the mutants were expressed at a significantly low level in the wild type tissue. (B) Tissues in which genes up-regulated in the mutants had low abundance, and tissues in which genes down-regulated in the mutants had high abundance. Colors in the top row indicate if genes up-regulated in the mutants were expressed at a significantly low level in the wild type tissue. Colors in the bottom row indicate if genes down-regulated in the mutants were expressed at a significantly high level. The key for tissue type abbreviations is: RT1: root, 7 days; RT2: root, 15 days; RT3: root, 17 days; MS1: shoot apex, vegetative plus young leaves; MS2: shoot apex, vegetative; MS3: shoot apex, transition; MS4: shoot apex, inflorescence; RL1: rosette leaf #4; RL2: rosette leaf #6; RL3: rosette leaf #8; RL4: rosette leaf #10; CL: cauline leaf; MP: mature pollen; SL1: silique, with seeds stage 3; SL2: silique, with seeds stage 4; SL3: silique, with seeds stage 5; SD1: seed, stage 6; SD2: seed, stage 7; SD3: seed, stage 8; SD4: seed, stage 9.

We also investigated whether miRNAs could cause the transcriptome of one tissue to resemble the transcriptome of another tissue. Using the tissue corrected distributional bias test (see materials and methods), we found that the up-regulated genes in miRNA biogenesis mutant inflorescences were preferentially expressed in wild type leaves and meristems (Figure 5A), and less expressed in pollen and seeds (Figure 5B). The down-regulated genes in miRNA biogenesis mutants were preferentially expressed in wild-type pollen, siliques and seeds (Figure 5B), and less expressed in roots, meristems and leaves (Figure 5A). Thus mutations in miRNA biogenesis genes cause the inflorescence transcriptome to resemble the leaf and meristem transcriptomes. In addition, the mutations make the inflorescence transcriptome less similar to mature pollen and seed transcriptomes.

## Discussion

### Transcriptional patterns of 21nt and 24nt miRNA-defective mutants

Across canonical miRNA biogenesis/transport mutants, the number of misexpressed, known miRNA targets did not correlate with the number of transcripts that changed in abundance. In addition, the number of known miRNA targets upregulated between mutants did not correlate with the number of mRNAs upregulated between mutants. Thus, we postulated that transcriptome differences amongst mutants are due to roles of the biogenesis proteins in processes outside of miRNA biogenesis and/or that a number of miRNA targets have not been identified. We examined an intersection of four mutant transcriptomes to identify novel miRNAs and miRNA targets.

Analysis of the miRNA mutant transcriptomes also showed that the pattern of transcriptional changes amongst mutants was not always consistent with the current model for miRNA biogenesis/transport [1], and that *dcl3* had little effect on the transcriptome. Transcripts upregulated in both *hyl1* and *hst* were the same almost two-times more often than the transcripts that changed in other *hyl1* and *hst* comparisons, and a relatively low number of transcripts were shared between *dcl1* and *hyl1*. Of the 930 transcripts upregulated in *hyl1*, 65% were upregulated in *hst15*, and of the 1,995 transcripts upregulated in *hst15*, 30% were found in *hyl1* (Figure 2A–2B). In *Arabidopsis*, RISC loading may occur both in the nucleus and in the cytoplasm [39]. One could envision HYL1 shuttling a subset of mature miRNAs to HST for export while other miRNA duplexes are integrated into the nuclear RISC. However, of the 37 known miRNA targets up-regulated in *hyl1* and of the 29 known miRNA targets upregulated in *hst*, 18 were shared (Figure S3), a number similar to other mutant pairs. As mentioned above, the role of HST as an exportin involved for miRNA transport has not been confirmed [13], [26]; we suggest that HYL may interact with HST in the nucleus to facilitate mature miRNA processing. In contrast, we had expected the highest overlap of transcript changes between *dcl1* and *hyl1* because miRNA targets accumulate less in *hyl1* than *dcl1* [12], and HYL1 interacts with DCL1 to promote the precise processing and efficient generation of miRNAs [4]. Only eight percent of *dcl1* transcripts overlapped with *hyl1*, and only 35% of *hyl1* transcripts overlapped with *dcl1* (Figure 2A). The hypomorphic *dcl1* mutant used in this study, *dcl1-7*, has a mutation in the helicase domain [25]. We considered the possibility that *dcl1-7* could still process HYL1 dependent miRNAs. However, *dcl1-7* has been shown to fail to process miR163 which is impaired in *hyl1* [3], and the number of shared known miRNA targets among up-regulated genes (24) is higher between *dcl1* and *hyl1* than between *dcl1* and other mutants (Figure S3). The function of HYL1 in miRNA biogenesis is thus likely DCL1-dependent, and the genes affected in the *hyl1* mutant that did not overlap with *dcl1* likely reflect other roles for this protein. HYL1 was recently shown to bind to short interspersed element (SINE element) RNA and influence a variety of cellular processes [40]. Future work could examine the relationship amongst SINEs and the gene transcript abundance changes in the HYL1 mutant. As expected, the *dcl1* and *hen1* mutants had a high number of shared transcript changes [3], [41]. DCL3 generates 23 to 25nt miRNAs from a number of the same miRNA precursors processed by DCL1 and is also necessary to process a number of 23- to 25-nt repeat-associated siRNAs (ra-siRNA) associated with heterochromatin and DNA repeats [10], [42]. The *dcl3* mutant had a small effect on the transcriptome with 258 and 210 transcripts up-regulated and down-regulated, respectively. This result supports the hypothesis that long miRNAs processed by DCL3 are functionally inert [1], despite the fact they are developmentally regulated and conserved over time [6], and the result is consistent with the limited role of repeats in *Arabidopsis* gene regulation [43].

### The discovery of novel miRNAs

We hypothesized that a core set of 123 genes up-regulated among all the canonical miRNA mutants (Figure 2) would contain a number of novel miRNA targets. Transcription factors are known preferential, direct targets for miRNAs [18], and among the core set of up-regulated transcripts, only the “transcription factor activity” GO molecular function term was highly over-represented (Table 1). This result was consistent with the expectation that these genes contained a number of direct miRNA targets. Interestingly, only the “unknown molecular functions” term was significantly under-represented amongst up-regulated genes (Table 1). The small number of upregulated genes with an unknown molecular function suggests that miRNAs regulate genes that have a noticeable mutant phenotype and thus have a high chance of experimental characterization and discovery. We had also anticipated that the 123 genes would contain downstream miRNA targets that were involved in specific molecular functions or biological processes. Because only transcription factors were over-represented, we conclude that miRNA regulated downstream genes have a range of molecular functions and are involved in a number of biological processes.

By integrating a number of lines of evidence, we identified five novel mature miRNAs. Four were highly similar to previously characterized miRNAs. The novel mature miRNAs met criteria for the annotation of plant microRNAs (Table 2) [44]. The mature 20∼21 nt miRNAs are expressed (Table 3). As expected, the miRNA precursors contain extensive base-pairing, stable and conserved stem-loop structures. No asymmetric bulges exist between the miRNA and the opposite stem-arm in the stem-loop (Figure S5). The novel mature miRNAs have high complementarity with their predicted target sites and meet empirical sequence parameters for miRNA∶target recognition such as no mismatches at position 10 and 11 of the 5′ end of miRNAs and no more than one mismatch at positions 2–12 (Figure S6) [45]. MiRNA 3 targets a novel mRNA. The other four mature miRNAs target mRNAs that are known targets of other miRNAs. Three of these four novel miRNAs direct mRNA cleavage at novel positions (Figure S6). This study identified miRNAs using only inflorescence transcriptome data and conservative criteria: putative targets had to be misexpressed in all mutants, and targets and miRNAs could differ by at most three nucleotides. It is very likely that analyses of individual biogenesis mutants and other biogenesis mutant tissues using the same or more liberal criteria would identify additional novel miRNAs and miRNA targets.

As described above, miRNAs are processed from a miRNA duplex. Mature miRNAs guide RISC to silence target mRNAs, and the complementary miRNA* is gradually degraded [39], [42]. Interestingly, one miRNA gene encodes miRNAs 3 and 4 from the same pre-miRNA stem loop (Table 2, Figure 3). One miRNA targets *At2g40840*, an enzyme involved in the starch to sucrose transition. The complement targets CCAAT-binding transcription factors. Xue et al. [46] suggested that high levels of a miRNA* may suppress the function of a miRNA. Because we identified miRNAs based on the reduction of putative targets, both miRNAs identified here very likely function to cleave target mRNAs. This discovery is similar to previous reports in mammals in which RISC was able to cleave the antisense of *let-7* target genes when the miRNA/miRNA* duplex was provided [47]. Both strands of miRNA can also suppress the expression of mouse transcripts [48].

### miRNA regulated genes have tissue-biased expression

We found that both up- and down-regulated genes in the inflorescences of miRNA biogenesis mutants were highly expressed in wild-type inflorescences relative to other tissues (Figure 4). This observation suggests that miRNAs as a class spatially restrict target transcripts within tissues and/or affect target transcripts at a specific developmental time rather than eliminate transcripts from whole tissues [1]. Co-expression of a miRNA and its target is consistent with this interpretation. miR171 and its targets SCL6-III and SCL6-IV are highly expressed in inflorescences [17]. In rice, osa-miR160, osa-miR164 and osa-miR172 are co-expressed with their targets in root, leaf, seedling, endosperm and embryo [46].

Using the distributional bias test, we also found that genes up-regulated in the 21nt miRNA biogenesis mutants' inflorescences were preferentially expressed in leaves and meristems and less expressed in pollen and seeds (Figure 5A). Genes down-regulated within the mutants' inflorescences are significantly abundant in wild type pollen and seed and significantly low in meristems, roots and leaves (Figure 5B). Thus, miRNA mutants cause the inflorescence transcriptome to resemble leaf and meristem transcriptomes and to diverge from pollen and seed transcriptomes. A number of developmental and molecular observations are consistent with this observation. The *dcl1* mutant displays defects in embryo development, floral meristem identity, and delayed flowering [25], and *hen1* and *hyl1* have similar developmental defects as *dcl1* [11], [12], [25]. Meristem and auxin related genes accumulate in the *hyl1* mutant inflorescences [12]. SE, along with DCL and HYL1 functions in primary microRNA processing [5]. Some siliques from F1 progeny of *se* and *hyl1* contained the abortive seeds because the *se hyl1* double mutant is embryonically lethal [5]. Finally, overexpression of an *AP2* cDNA with mismatches to miR172 caused enlarged floral meristems and many whorls of petals or staminoid organs [29]. Despite these observations, it was not clear *a priori* that loss of miRNAs as a class would cause one tissue's transcriptome to resemble that of another because different miRNA∶target interactions are known to have opposite functions. For example, overexpression of miRNA156 in maize prolongs juvenile development while expression of miR172 promotes the transition to the adult phase of growth [30], [32]. It would be interesting to examine how miRNAs globally alter the transcriptomes in tissues other than the inflorescence.

In conclusion, analysis and integration of large mRNA profiling datasets, sRNA deep sequencing data, and computational predictions of conserved genomic stem-loop structures revealed novel mature miRNAs, novel miRNA targets, and miRNA-regulated developmental changes in gene expression in the model plant *Arabidopsis thaliana*. miRNAs have been identified in the important crop species maize [49], [50] and rice [46], [51]. It would be interesting to see if miRNAs in these diverse agriculturally important species also promote reproductive transcriptome changes.

## Materials and Methods

### Evaluating transcript abundance changes and tissue-biased expression

ATH1 microarray raw data (.cel files) from wild type and miRNA-defective mutants, *dcl1-7*, *dcl3-1*, *hen1-1*, *hyl1-2* and *hst15* were kindly provided by Dr. Carrington's lab. Plant growth conditions, RNA extractions, labeling and hybridization were described by Allen et al., [21]. Arrays were hybridized with cRNA derived from inflorescence RNA. We used data from three arrays from each mutant line and three wild type lines. We normalized data using gcRMA implemented in R [52]. The expression level of each gene was represented by the mean of the log2-transformed, normalized and median polished signal levels across each set of tissue replicates, and significant differences between mutant and wild type were determined using linear models in the Bioconductor LIMMA package with a Bayesian correction for standard error. Differences were deemed statistically significant with a false discovery rate (FDR) of 0.05. We defined core sets of genes as those genes whose transcript abundance was higher or lower across all the canonical miRNA mutants.

To determine the tissue abundance of genes that were misexpressed in miRNA mutants, we obtained Affymetrix ATH1 data described by Schmid et al. [38]. We selected expression data from 23 of 79 tissues excluding similar tissue types to avoid over-representation in statistical analyses. Tissue descriptions and abbreviations are given in Table S2. Each gene's expression levels were ranked over all 23 tissues. We determined if core sets of genes had high ranks or low ranks within a tissue compared to the ranks of a reference group, the genes not included within the core sets. A gene in a core set with a high rank within a tissue meant that the gene's transcript abundance was high in the tissue relative to other tissues.

The Komolgorov-Smirnov (KS) test was used to identify tissues in which core sets of up-regulated or down-regulated genes had significantly high or low abundances. The mutant transcripts were sampled from the inflorescence, and thus *a priori* may have had high ranks in tissues with transcriptomes more like the inflorescence than other tissues. To avoid this bias, we compared the abundance of miRNA-regulated genes across tissues with genes that have the same expression ranks in the inflorescence. We randomly chose sets of genes which had the same expression ranks as genes in the core sets in the inflorescence 1,000 times. For each gene set, we applied the KS test. The core sets' observed KS p values were compared with the KS p values from the permuted data set to determine the significance of the core set KS value. We named this approach the tissue corrected distributional bias test. A core set had significantly higher or lower transcript levels in the tissue of interest relative to other tissues if the P-value was less than 0.01.

Computational analyses were performed on a high performance computing “cluster of clusters”, the Shared Hierarchical Academic Research Computing NETwork (SHARCNET). We utilized the cornfish, narwhal, and whale clusters each with 14, 1,074 and 3,082 processors respectively. SHARCNET enabled rapid computations and the execution of some analyses that required large memory configurations (up to 16GB per processor).

### Identifying novel miRNAs and corresponding targets

Predicted *A. thaliana* genomic and mRNA sequences were retrieved from GenBank RefSeqs (accessions: NC_003070.6, NC_003071.4, NC_003074.5, NC_003075.4, and NC_003076.5). A set of 2,585 *Arabidopsi*s 20mers (*At*Set3) which are located in potential hairpin structures typical of miRNA precursors and are conserved in rice was kindly provided [18]. Putative miRNA sequences generated by 454 sequencing were downloaded from NCBI. This data set contained 1,984 unique sRNAs derived from *rdr2* inflorescence tissue which is enriched for miRNAs [10]. We compared transcripts whose abundance increased among mutants to both the sRNAs and the *At*Set3 list of sequences to identify direct miRNA targets. First, the 1,984 sRNAs were blasted against the mRNAs of up-regulated genes. Three or fewer mismatches between a sRNA and an mRNA were considered a hit. Second, we determined the presence of the matched sRNAs within *At*Set3, allowing only perfect matches between the sRNAs and *At*Set 3 sequences. To visualize hairpin structures, ∼500 nt genomic sequences centred on each matched sRNA were retrieved from the GenBank RefSeq records. With those genomic sequences, RNAfold implemented in ViennaRNA-1.8.1 [36] was used to predict the secondary structure. The RNAfold-predicted secondary structure was tested for miRNA hairpins and evaluated for a miRNA and miRNA* duplex by MIRcheck.

## Supporting Information

Figure S1 The frequency of known miRNA targets amongst genes up- and down-regulated in the miRNA biogenesis mutants. Known miRNA targets were highly represented amongst up-regulated genes.
(0.20 MB TIF)

Figure S2 Histograms of log-fold changes of known miRNA target transcripts represented in significantly up-regulated genes among miRNA biogenesis mutants.
(0.22 MB TIF)

Figure S3 Three-way Venn diagrams showing the overlap of known miRNA targets up-regulated within miRNA biogenesis mutants *dcl1-7*, *hen1-1*, *hyl1-2* and *hst15*.
(0.22 MB TIF)

Figure S4 A plot of Pearson's Chi-squared test residuals of GOslim molecular function classes for genes up-regulated in canonical miRNA mutants compared to genes that were not up-regulated in the mutants. DRB combined DNA or RNA binding, nucleic acid binding, and nucleotide binding; HA, hydrolase activity; KA, kinase activity; OB, other binding; OEA, other enzyme activity; OMF, other molecular functions; PB, protein binding; RBA, receptor binding or activity; SMA, structural molecule activity; Ta, transferase activity; TA, transporter activity; TFA, transcription factor activity; UMF, unknown molecular functions.
(0.11 MB TIF)

Figure S5 Predicted hairpin structures of the miRNA genes that give rise to five mature miRNAs. All the hairpin structures are predicted by the RNAfold program. Mature miRNA sequences in each family recovered from 454 sRNA sequence data are represented by vertical red bars located either on the 5-prime or the 3-prime arm of the precursor.
(2.00 MB TIF)

Figure S6 Watson-Crick pairing between novel mature miRNAs and predicted miRNA targets. There were no mismatches at positions 10 and 11 from the 5′ end of miRNAs with nucleotides in miRNA targets (red).
(0.28 MB TIF)

Table S1 Goslim categories in biological process (BP) of differentially expressed genes and non-differentially expressed genes in *dcl1-7*, *hen1-1*, *hyl1-2* and *hst15*.
(0.07 MB DOC)

Table S2 Sample names and descriptions for ATH1 microarray data.
(0.04 MB DOC)
