# Supplementary figures and images for: Identification of Novel miRNAs and miRNA Dependent Developmental Shifts of Gene Expression in Arabidopsis thaliana
Source: PLoS One. 2010 Apr 13;5(4):e10157. doi: 10.1371/journal.pone.0010157 (PMC2854152; doi:10.1371/journal.pone.0010157)

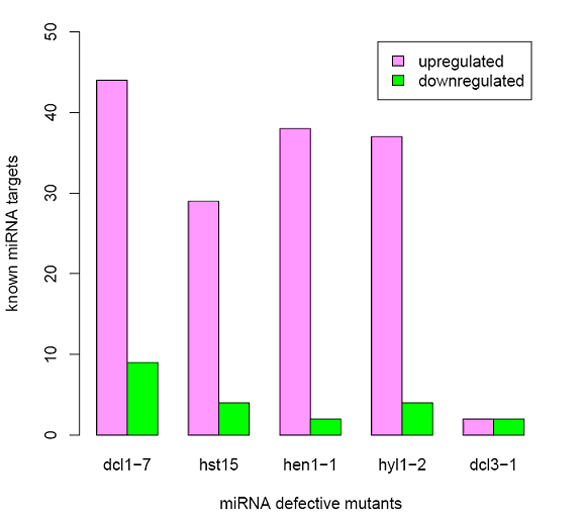

Supplement: Figure S1 — The frequency of known miRNA targets amongst genes up- and down-regulated in the miRNA biogenesis mutants. Known miRNA targets were highly represented amongst up-regulated genes. (0.20 MB TIF) [file pone.0010157.s001.tif]

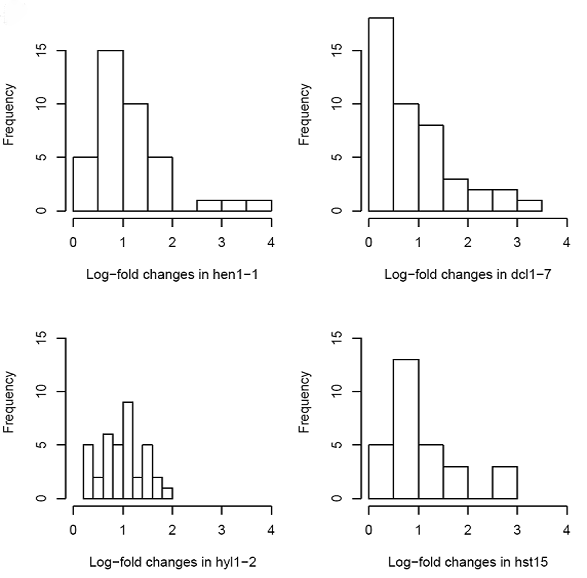

Supplement: Figure S2 — Histograms of log-fold changes of known miRNA target transcripts represented in significantly up-regulated genes among miRNA biogenesis mutants. (0.22 MB TIF) [file pone.0010157.s002.tif]

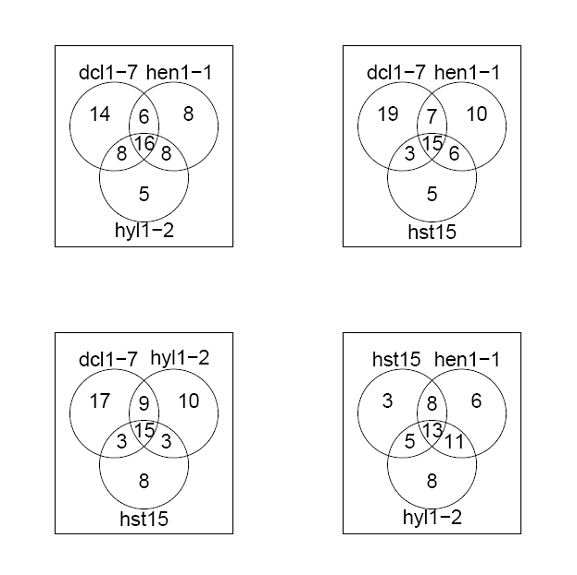

Supplement: Figure S3 — Three-way Venn diagrams showing the overlap of known miRNA targets up-regulated within miRNA biogenesis mutants dcl1-7, hen1-1, hyl1-2 and hst15. (0.22 MB TIF) [file pone.0010157.s003.tif]

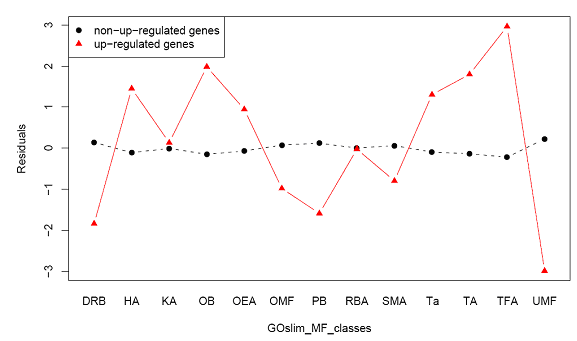

Supplement: Figure S4 — A plot of Pearson's Chi-squared test residuals of GOslim molecular function classes for genes up-regulated in canonical miRNA mutants compared to genes that were not up-regulated in the mutants. DRB combined DNA or RNA binding, nucleic acid binding, and nucleotide binding; HA, hydrolase activity; KA, kinase activity; OB, other binding; OEA, other enzyme activity; OMF, other molecular functions; PB, protein binding; RBA, receptor binding or activity; SMA, structural molecule activity; Ta, transferase activity; TA, transporter activity; TFA, transcription factor activity; UMF, unknown molecular functions. (0.11 MB TIF) [file pone.0010157.s004.tif]

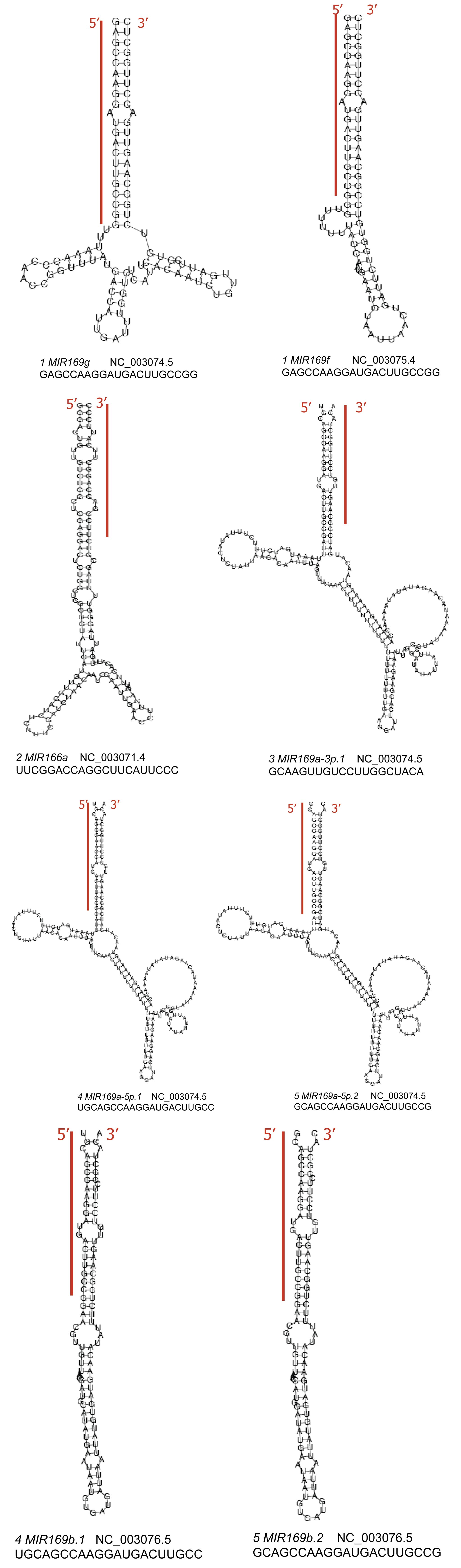

Supplement: Figure S5 — Predicted hairpin structures of the miRNA genes that give rise to five mature miRNAs. All the hairpin structures are predicted by the RNAfold program. Mature miRNA sequences in each family recovered from 454 sRNA sequence data are represented by vertical red bars located either on the 5-prime or the 3-prime arm of the precursor. (2.00 MB TIF) [file pone.0010157.s005.tif]

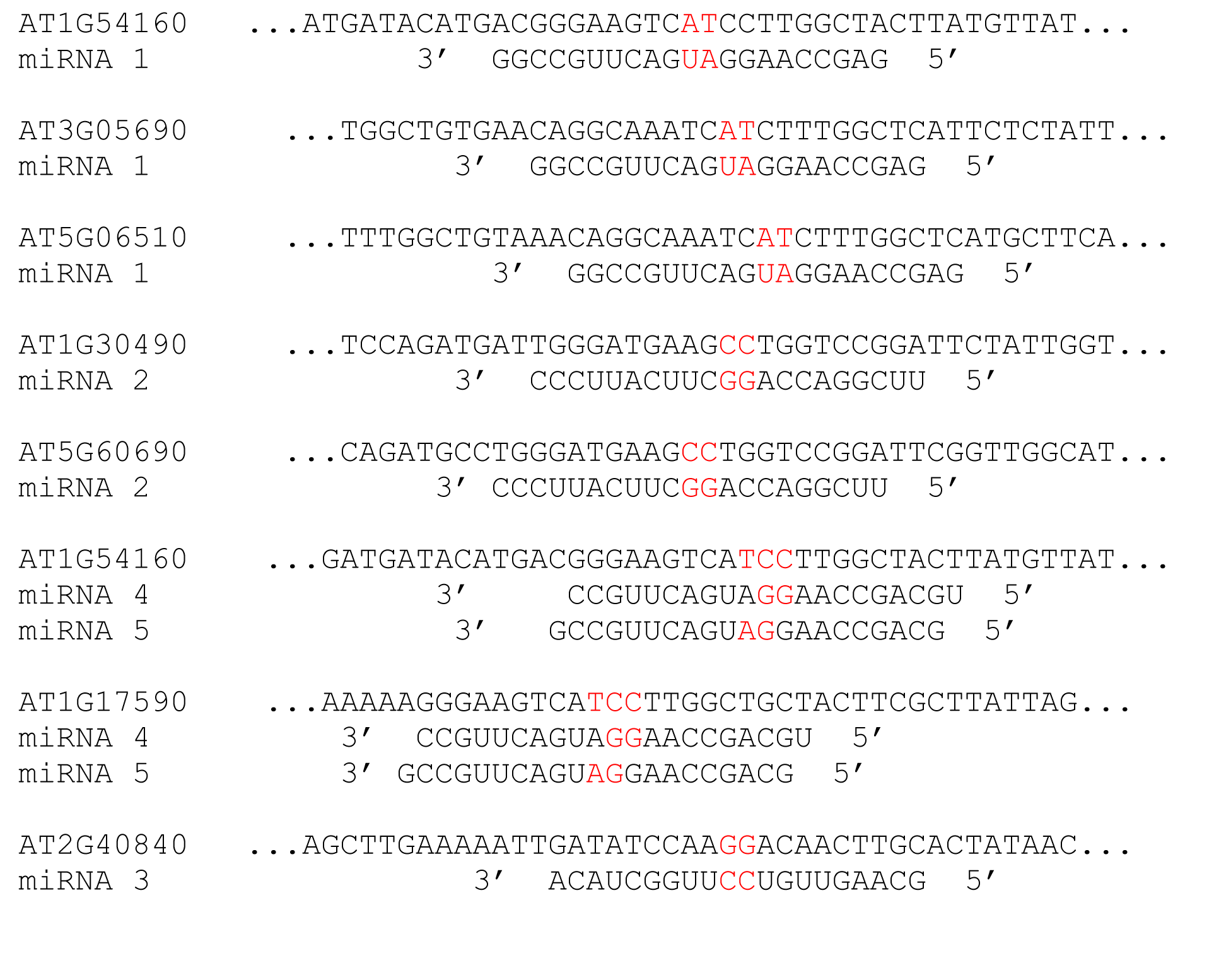

Supplement: Figure S6 — Watson-Crick pairing between novel mature miRNAs and predicted miRNA targets. There were no mismatches at positions 10 and 11 from the 5′ end of miRNAs with nucleotides in miRNA targets (red). (0.28 MB TIF) [file pone.0010157.s006.tif]
