# Supplementary material for: Identification of Novel miRNAs and miRNA Dependent Developmental Shifts of Gene Expression in Arabidopsis thaliana
Source: PLoS One. 2010 Apr 13;5(4):e10157. doi: 10.1371/journal.pone.0010157 (PMC2854152; doi:10.1371/journal.pone.0010157)
Supplement: Table S1 — Goslim categories in biological process (BP) of differentially expressed genes and non-differentially expressed genes in dcl1-7, hen1-1, hyl1-2 and hst15. (0.07 MB DOC) [file pone.0010157.s007.doc]

| **Table S1.** Goslim categories in biological process (BP) of differentially expressed genes and non-differentially expressed genes in *dcl1-7, hen1-1, hyl1-2 and hst15* | | | | | | | | | | | | | | | |
| --- | --- | --- | --- | --- | --- | --- | --- | --- | --- | --- | --- | --- | --- | --- | --- |
|  | Non-up-regulated genes | | |  | Up-regulated genes | | |  | Down-regulated genes | | |  | Non-down-regulated genes | | |
| Goslim categories | Genes |  | Proportion (%) |  | Genes |  | Proportion (%)* |  | Genes |  | Proportion (%)^†^ |  | Genes |  | Proportion (%) |
| DNA or RNA metabolism | 251 |  | 0.76 |  | 0 |  | 0.00 |  | 0 |  | 0.00 |  | 251 |  | 0.76 |
| cell organization and biogenesis | 1220 |  | 3.70 |  | 7 |  | 3.03 |  | 7 |  | 4.22 |  | 1220 |  | 3.69 |
| developmental processes | 1098 |  | 3.33 |  | 10 |  | 4.33 |  | 5 |  | 3.01 |  | 1103 |  | 3.34 |
| electron transport or energy pathways | 599 |  | 1.82 |  | 1 |  | 0.43 |  | 3 |  | 1.81 |  | 597 |  | 1.81 |
| other biological processes | 867 |  | 2.63 |  | 9 |  | 3.90 |  | 14 |  | 8.43 |  | 862 |  | 2.61 |
| other cellular processes | 6530 |  | 19.81 |  | 53 |  | 22.94 |  | 27 |  | 16.27 |  | 6556 |  | 19.85 |
| other metabolic processes | 6941 |  | 21.06 |  | 55 |  | 23.81 |  | 40 |  | 24.10 |  | 6956 |  | 21.06 |
| protein metabolism | 2724 |  | 8.26 |  | 12 |  | 5.19 |  | 8 |  | 4.82 |  | 2728 |  | 8.26 |
| response to abiotic or biotic stimulus | 1130 |  | 3.43 |  | 15 |  | 6.49 |  | 10 |  | 6.02 |  | 1135 |  | 3.44 |
| response to stress | 1329 |  | 4.03 |  | 13 |  | 5.63 |  | 8 |  | 4.82 |  | 1334 |  | 4.04 |
| signal transduction | 793 |  | 2.41 |  | 4 |  | 1.73 |  | 3 |  | 1.81 |  | 794 |  | 2.40 |
| transcription | 1333 |  | 4.04 |  | 14 |  | 6.06 |  | 6 |  | 3.61 |  | 1341 |  | 4.06 |
| transport | 1426 |  | 4.33 |  | 13 |  | 5.63 |  | 4 |  | 2.41 |  | 1435 |  | 4.34 |
| unknown biological processes | 6722 |  | 20.39 |  | 25 |  | 10.82 |  | 31 |  | 18.67 |  | 6716 |  | 20.33 |
| *Proportions in the 14 Goslim BP categories differed significantly from those for non-up-regulated genes (Fisher's exact test, P=9x10^-4^). | | | | | | | | | | | | | | | |
| ^†^Proportions in the 14 Goslim BP categories did not differ siginificantly from those for non-down-regulated genes (Fisher's exact test, P=2x10^-2^). | | | | | | | | | | | | | | | |
