# Supplementary material for: Identification of Novel miRNAs and miRNA Dependent Developmental Shifts of Gene Expression in Arabidopsis thaliana
Source: PLoS One. 2010 Apr 13;5(4):e10157. doi: 10.1371/journal.pone.0010157 (PMC2854152; doi:10.1371/journal.pone.0010157)
Supplement: Table S2 — Sample names and descriptions for ATH1 microarray data. (0.04 MB DOC) [file pone.0010157.s008.doc]

| **Table S2.** Sample names and descriptions for ATH1 microarray data | |
| --- | --- |
| Abbreviations* | Tissues |
| RT1 | root, 7 days |
| RT2 | root,15 days |
| RT3 | root, 17 days |
| MS1 | shoot apex, vegetative + young leaves |
| MS2 | shoot apex, vegetative |
| MS3 | shoot apex, transition (before bolting) |
| MS4 | shoot apex, inflorescense (after bolting) |
| RL1 | rosette leaf #4, 17 days |
| RL2 | rosette leaf #6, 17 days |
| RL3 | rosette leaf #8, 17 days |
| RL4 | rosette leaf #10, 17 days |
| CL | cauline leaf |
| MP | mature pollen |
| FL1 | flower, stage 9, the petals become stalked at base |
| FL2 | flower, stage 10/11, stigmatic papillae cover the stigma |
| FL3 | flower, stage 12, the petals reach the length of the long medial stamens |
| SL1 | silique, with seeds stage 3; mid globular to early heart embryo |
| SL2 | silique, with seeds stage 4; early to late heart embryo |
| SL3 | silique, with seeds stage5; late heart to mid torpedo embryo |
| SD1 | seed, stage 6; mid to late torpedo embryos |
| SD2 | seed, stage 7; late torpedo to early walking-stick embryos |
| SD3 | seed, stage 8; walking-stick to early curled-cotyledons embryo |
| SD4 | seed, stage 9; curled-cotyledons to early green-cotyledons embryo |
| *Samples were selected from the plant development dataset described by Schmid et al. (Schmid et al. 2005) | |
